# Three distinct trajectories of early oral feeding ability in preterm infants and their associated clinical factors

**DOI:** 10.3389/fped.2026.1924274

**Published:** 2026-10-20

**Authors:** Xiuru Tang, Yanan Zhu, Yumei Wu, Shiyan Sun, Minmin Zhou, Xiurong Yu

**Affiliations:** 1 Department of Neonatal Intensive Care Unit, Affiliated Hospital of Shandong Medical and Pharmaceutical University, Binzhou, Shandong, China; 2 School of Nursing, Shandong Medical and Pharmaceutical University, Binzhou, Shandong, China; 3 Department of Nursing, Affiliated Hospital of Shandong Medical and Pharmaceutical University, Binzhou, Shandong, China

**Keywords:** associated factors, latent class growth modeling, longitudinal study, oral feeding ability, preterm infants, trajectories

## Abstract

**Background:**

Globally, approximately 13.4 million infants are born preterm each year, and difficulties during the transition to oral feeding are common among preterm infants during NICU hospitalization. Existing studies on oral feeding ability in preterm infants are predominantly cross-sectional, failing to identify underlying heterogeneity. This study used latent class growth modeling (LCGM) to identify trajectories of oral feeding ability during the first 14 days after oral feeding initiation and examine clinical characteristics associated with trajectory membership.

**Methods:**

This prospective cohort study enrolled preterm infants (gestational age 28–32 weeks) from a tertiary hospital neonatal intensive care unit (NICU) between May 2025 and April 2026. Oral feeding ability was assessed at Days 1, 3, 7, 10, and 14 using the Oral Feeding Ability Scale for Preterm Infants. LCGM identified trajectories; multinomial logistic regression examined factors associated with trajectory membership.

**Results:**

Of the 245 enrolled infants, 213 (86.9%) completed all five scheduled assessments. Scores increased significantly from 22.03 ± 2.95 on Day 1 to 34.41 ± 3.67 on Day 14 (*p* < 0.001). Three trajectories were identified: high-start rapid-rise (31.5%), moderate-start accelerated catch-up (44.6%), and low-start slow-rise (23.9%). Variables associated with trajectory membership included gestational age, birth weight, postmenstrual age (PMA) at oral feeding initiation, mechanical ventilation, illness severity (Neonatal Medical Index, NMI), and pre-feeding behavioral state.

**Conclusions:**

Significant heterogeneity was observed in the developmental trajectories of early oral feeding ability. In this exploratory single-center cohort of formula-fed preterm infants, clinical characteristics were associated with trajectory membership; these findings should be considered associative and hypothesis-generating rather than predictive.

## 1 Introduction

Globally, approximately 13.4 million infants are born preterm each year, accounting for roughly 10% of all live births (1). Although advances in neonatal intensive care have markedly improved survival rates, preterm birth and its associated sequelae remain leading contributors to mortality among children under five years of age worldwide (2). Adequate nutritional support is essential for improving outcomes in preterm infants, with oral feeding being the preferred method (3). Safe and effective transition to full oral feeding is a critical prerequisite for neonatal intensive care unit (NICU) discharge (4); failure to achieve this milestone prolongs hospital stays, increases healthcare resource utilization, and imposes additional burdens on families (5).

Hospitalized preterm infants may experience oral feeding difficulties characterized by weak sucking, poor suck–swallow–breathe coordination, oxygen desaturations, and bradycardia (6, 7). Problematic feeding is also highly prevalent in early childhood, with a meta-analysis reporting an overall prevalence of 42% among prematurely born children (8), underscoring the need for timely monitoring and intervention. Oral feeding in preterm infants requires coordinated neurological, gastrointestinal, and respiratory function; however, immature physiological development, assisted ventilation, and prolonged tube feeding may hinder the acquisition of oral feeding competence (9).

Existing research has largely relied on cross-sectional designs, examining whether infants achieve full oral feeding by specific postmenstrual ages or within fixed timeframes (10, 11). While these studies provide useful benchmarks for discharge readiness, they are unable to capture the non-linear, progressive maturation of oral feeding ability or reveal distinct subgroups with heterogeneous developmental patterns. Latent class growth modeling (LCGM) is a person-centered approach that classifies individuals into unobserved subgroups based on their longitudinal trajectories, thereby uncovering distinct patterns of change that aggregate analyses may conceal (12).

Therefore, this study aimed to: (1) identify distinct trajectories of oral feeding ability during the first 14 days after oral feeding initiation in preterm infants using LCGM; (2) examine clinical and developmental characteristics associated with latent subgroup membership; and (3) identify clinical characteristics associated with trajectory membership and generate hypotheses for future research.

## 2 Materials and methods

### 2.1 Design and sample

This prospective cohort study was conducted in the neonatal intensive care unit (NICU) at the Affiliated Hospital of Shandong Medical and Pharmaceutical University in China. Preterm infants enrolled in this study were admitted between May 2025 and April 2026 and were assessed at days 1, 3, 7, 10, and 14 after oral feeding initiation (five measurement points in total). The 14-day observation period was selected based on clinical evidence that preterm infants require approximately 7.5 ± 6.6 days to transition to full oral feeding (13), suggesting that a 14-day window captures the majority of feeding trajectories while remaining feasible for prospective data collection.

The study applied inclusion and exclusion criteria for participant selection. The inclusion criteria were as follows: (1) gestational age at birth between 28 and 32 weeks; (2) birth weight < 2500 g; (3) admission to the NICU within 24 h of birth with stable vital signs; (4) initial feeding via nasogastric or orogastric tube, or a combination of tube feeding and parenteral nutrition; (5) infants received the uniformly prepared preterm formula used by the unit. The exclusion criteria included (1) congenital anomalies, including oral malformations, cleft lip, cleft palate, congenital heart disease, or intestinal atresia and (2) major neonatal complications, including intraventricular hemorrhage, necrotizing enterocolitis, or sepsis. Enrollment was restricted to formula-fed infants to reduce variation in feeding type; this was a study-design criterion rather than the unit's routine feeding practice. Because individual intake was not recorded, this restriction did not establish comparable nutritional exposure.

Sample size was estimated using G*Power 3 (Heinrich Heine University, Düsseldorf, Germany) for repeated-measures longitudinal designs (14). With *α* = 0.05, statistical power (1−*β*) = 0.80, an expected effect size f = 0.25, and five measurement points, the minimum required sample size was 193. Accounting for a 10%–20% attrition rate, the target sample size was 212–231.

### 2.2 Feeding strategy during hospitalization

1. Initiation and goals of enteral and parenteral nutrition: According to the unit's standardized feeding protocol, enteral nutrition was started as early as possible within 24 h after birth in infants who were hemodynamically stable and had no contraindications. Feedings were administered as scheduled intermittent bolus feeds via nasogastric tube every 2–3 h. The study formula was Zao Rui Neng En 400 g infant formula for special medical purposes [registration no. TY20185006; manufacturer and registration holder: NESTLE NEDERLAND B.V., Laan 110, 8071 JC Nunspeet, The Netherlands; domestic representative: Nestlé (China) Ltd., Beijing, China]. It was prepared by mixing 16.1 g of formula powder with 90 mL of water; the prepared formula provided approximately 80.1 kcal and 2.32 g of protein per 100 mL (protein-to-energy ratio, 2.90 g/100 kcal). The daily increase in formula volume did not exceed 20 mL/kg. The target for full enteral feeding was 150–180 mL/(kg·d), maintained for more than 48 h. Feeding intolerance was considered when clinical signs such as vomiting and/or abdominal distension required interruption, reduction, or withholding of enteral feeding. Gastric residual volume was not measured routinely; when such clinical signs were present, a gastric residual volume exceeding 50% of the previous feeding volume was used as an additional indicator. The enteral energy target was 115–140 kcal/(kg·d), with a protein intake of 3.5–4.0 g/(kg·d) and a protein-to-energy ratio of 2.8–3.6 g/100 kcal. These nutrient values describe the prescribed formula and protocol targets; they do not represent measured individual intake. Because individual feeding adjustments were made according to clinical tolerance, individual daily intakes and deviations from the prescribed volumes were not recorded in the study dataset. Parenteral nutrition (PN) was initiated in infants in whom adequate enteral nutrition was unlikely to be established within 48–72 h, or in whom enteral feeding could not meet nutritional requirements. PN was gradually reduced as enteral nutrition (EN) increased, and was discontinued when EN reached 120–140 mL/(kg·d) or provided approximately 110 kcal/(kg·d), with good feeding tolerance and steady weight gain.
2. Initiation and implementation of oral feeding: When the infant reached a postmenstrual age (PMA) of at least 32 weeks, had stable vital signs, and had been weaned from mechanical ventilation for more than 48 h, the attending physician issued the order to initiate oral feeding, with the order taking effect at the next scheduled feeding. Feedings were scheduled every 2–3 h. At the daily morning assessment, the volume of each oral feed was increased by 2–3 mL compared with the previous day if the infant had no vomiting or milk regurgitation. The responsible nurse used the attempt-and-observe feeding approach. The infant was first placed in a side-lying position with the head elevated, keeping the head and hips aligned and providing adequate support for the head, neck, and back. The nipple was gently touched to one corner of the infant's mouth to elicit the rooting reflex and was then placed into the mouth for feeding. Oral feeding was attempted even when the infant was drowsy or asleep. If sucking and swallowing could not be elicited, the feeding was given by nasogastric tube. During oral feeding, once vomiting, decreased heart rate, or oxygen saturation below 85% occurred, feeding was stopped immediately, and the remaining volume was given by nasogastric tube.

### 2.3 Ethical considerations

This study was approved by the Ethics Committee of the Affiliated Hospital of Shandong Medical and Pharmaceutical University (Approval No.: KYLL-275) and was conducted in accordance with the Declaration of Helsinki. Written informed consent was obtained from parents or legal guardians of all enrolled infants prior to data collection. Parents or legal guardians were informed of their right to withdraw at any time.

### 2.4 Data collection

Data were collected from May 2025 to April 2026. Within 24 h of NICU admission, electronic medical records were reviewed to confirm eligibility. Parents or legal guardians of eligible infants were informed of the study objectives and of their right to withdraw at any time. Written informed consent was obtained prior to enrollment. Baseline demographic and clinical characteristics were collected using a self-designed questionnaire within 24 h of admission.

Oral feeding ability was assessed at five time points: Day 1 (T1), Day 3 (T2), Day 7 (T3), Day 10 (T4), and Day 14 (T5) after the initiation of oral feeding. At each time point, the Oral Feeding Ability Scale for Preterm Infants was administered during three daily feeding sessions, and the mean of the three session-level scores was used as the daily oral feeding ability score. A session was scored when all four dimensions were evaluable. When sucking or swallowing was not elicited, a score of 0 was assigned to each affected dimension according to the scale scoring rules; the remaining dimensions were scored according to observed performance. Each session total was included in the daily mean of the three sessions, and no session was excluded solely because sucking or swallowing was absent. Illness severity was assessed at discharge using the Neonatal Medical Index (NMI).

### 2.5 Measurement instruments

#### 2.5.1 Socio-demographic and clinical characteristics

The questionnaire was designed by the research team following a literature review and comprises two sections: demographic information and feeding information. Demographic information includes: gestational age at birth, birth weight, sex, 1-minute Apgar score, mode of delivery, twin pregnancy, maternal age, and maternal perinatal history; feeding information includes: postmenstrual age (PMA) at initiation of oral feeding, weight at initiation of oral feeding, and whether mechanical ventilation was required. Respiratory data were limited to a binary variable indicating whether mechanical ventilation was ever required; duration of mechanical ventilation, bronchopulmonary dysplasia (BPD), and respiratory support status at oral feeding initiation were not collected.

#### 2.5.2 Oral feeding ability scale for preterm infants

The Chinese version of the Oral Feeding Ability Scale for Preterm Infants was used to evaluate oral feeding ability. This scale was adapted from Thoyre's Early Feeding Skills (EFS) scale for use in the Chinese clinical context (15). It comprises four dimensions: pre-feeding condition (7 items), sucking ability (5 items), swallowing ability (4 items), and physiological stability during feeding (7 items), totaling 23 items. Each item is scored on a 3-point scale (0, 1, 2), with total scores ranging from 0 to 46; higher scores indicate better oral feeding ability. Internal consistency was acceptable, with Cronbach's *α* = 0.784 (16). No officially validated cut-off values or categorical classifications of feeding ability have been established for this scale; the total score was therefore analyzed as a continuous variable in the present study.

#### 2.5.3 Anderson behavioral state scale

Developed by Anderson et al. (17), the Anderson Behavioral State Scale assesses 12 specific behavioral patterns in preterm infants. Trained observers rate the infant's state after a 30-second continuous observation period (18). Inter-rater reliability is reported to range from 0.80 to 0.90 (19). This scale has been widely used to assess behavioral states in preterm infants. Pre-feeding behavioral state calculation: At each time point, the infant's behavioral state was assessed immediately prior to each of the three feeding sessions. The mean of the three session scores was calculated to obtain the daily pre-feeding behavioral state score for that time point. Subsequently, the mean of these daily scores across the five time points was computed as the overall pre-feeding behavioral state score for each infant. Because no externally validated cut-off values are available for the ABSS summary score, the 12 states were collapsed *a priori* by the research team into three operational categories: low arousal (1–5), moderate arousal (6–7), and high arousal (8–12). These categories were used for clinical interpretability and do not redefine the original ABSS states.

#### 2.5.4 Neonatal medical index

Developed by Korner et al. (20), the Neonatal Medical Index comprehensively assesses illness severity in newborns based on seven factors: birth weight; duration of respiratory support and oxygen requirements; apnea or bradycardia and its treatment; severity of patent ductus arteriosus; intracranial hemorrhage and white matter damage; hyperbilirubinemia requiring phototherapy or exchange transfusion; and major surgery or neurological complications. Infants are classified into five grades (Grade I to Grade V), with Grade I representing a stable condition and Grade V representing severe illness with complications. The Neonatal Medical Index has been associated with later developmental outcomes in preterm infants, and higher grades on the index have been associated with less favorable neurobehavioral functions requiring vigor and strength (21). Although the NMI was developed more than three decades ago, it remains widely used in contemporary preterm cohorts and was selected as a composite measure of neonatal illness severity (22, 23).

### 2.6 Quality control

Before data collection, all assessors completed standardized training and pre-study calibration on the Oral Feeding Ability Scale for Preterm Infants and the Anderson Behavioral State Scale. At each time point (Days 1, 3, 7, 10, and 14 after oral feeding initiation), trained assessors who were single-blind to the study hypotheses scored both instruments during three scheduled feeding sessions.

### 2.7 Data analysis

All data were analyzed using IBM SPSS Statistics 25.0 (IBM Corp., Armonk, NY, USA) and Mplus 8.0 (Muthén & Muthén, Los Angeles, CA, USA), with a two-tailed significance level of *α* = 0.05. Continuous variables with a normal distribution were described using mean (standard deviation); non-normally distributed continuous variables were presented as median (lower quartile, upper quartile) and analyzed using the Kruskal–Wallis H test. Categorical data were presented as frequencies (percentages) and analyzed using the chi-squared test or Fisher's exact test, as appropriate.

Generalized Estimating Equations (GEE) were performed in SPSS 25.0 to examine the overall trend of oral feeding ability over the five assessment points, accounting for within-subject correlation of repeated measurements. The dependent variable was the oral feeding ability score, and the independent variable was time (Day 1, 3, 7, 10, 14). An exchangeable correlation structure was specified, and model-based standard errors were used. Time was treated as a categorical variable to capture non-linear change patterns across the 14-day observation period.

Latent class growth modeling (LCGM) was subsequently performed using Mplus 8.0 (Muthén & Muthén, Los Angeles, CA, USA) to identify heterogeneous trajectories of oral feeding ability in preterm infants during the first 14 days after oral feeding initiation. Unlike standard growth curve modeling, LCGM assumes that individuals belong to distinct unobserved subpopulations with different developmental trajectories, with zero within-class variance (i.e., fixed intercepts and slopes within each latent class). One- to five-class models were fitted sequentially. The final solution was selected based on model fit indices, class separation, parsimony, clinical interpretability, and class size. The following indices were used: Akaike Information Criterion (AIC), Bayesian Information Criterion (BIC), sample-size adjusted BIC (aBIC), entropy, the Vuong–Lo–Mendell–Rubin likelihood ratio test (VLMR), and the bootstrap likelihood ratio test (BLRT). Lower AIC, BIC, and aBIC values indicate better model fit. A significant *p*-value (< 0.05) for VLMR and BLRT indicates that the k-class model fits significantly better than the (k-1)-class model. Entropy ranges from 0 to 1, with values > 0.80 indicating adequate class separation.

After trajectory identification, latent class membership was exported as a categorical dependent variable. Univariate analyses were first performed to compare baseline characteristics across the identified trajectories. Continuous variables were analyzed using one-way analysis of variance or the Kruskal–Wallis H test, and categorical variables were analyzed using the chi-squared test or Fisher's exact test, as appropriate. Variables demonstrating a univariate association with trajectory membership (*p* < 0.05) were entered into a multinomial logistic regression model performed in SPSS 25.0 to limit model complexity. The trajectory with the most favorable oral feeding ability profile was designated as the reference category. Multicollinearity among candidate variables was assessed using the variance inflation factor (VIF), with VIF ≥ 10 indicating serious multicollinearity.

## 3 Results

### 3.1 Characteristics of preterm infants

Of 245 enrolled infants, 27 were excluded due to worsening condition, transfer, or treatment withdrawal before oral feeding initiation, and 5 were lost during follow-up. The demographic and clinical characteristics of 213 preterm infants who completed all five assessments are summarized in Table 1, stratified by three oral feeding ability trajectory classes. The final sample had a mean gestational age of 30.7 weeks (SD 1.00). Overall, 102 (47.9%) were male and 111 (52.1%) were female; 195 (91.5%) had 1-minute Apgar scores ≥ 7 and 18 (8.5%) had scores < 7; 157 (73.7%) received mechanical ventilation and 56 (26.3%) did not; 52 (24.4%) were in a low-arousal state, 95 (44.6%) in a moderate-arousal state, and 66 (31.0%) in a high-arousal state before feeding; 41 (19.2%) were delivered vaginally and 172 (80.8%) by cesarean section; 16 (7.5%) were from twin pregnancies and 197 (92.5%) were singletons; 153 (71.8%) had illness severity NMI < 4 and 60 (28.2%) had NMI ≥ 4; 140 (65.7%) had maternal perinatal history and 73 (34.3%) did not.

**Table 1:** Demographic, clinical, and feeding-related characteristics of 213 preterm infants stratified by early oral feeding ability trajectory class.

Variables	Class 1(*n* = 67)	Class 2(*n* = 95)	Class 3(*n* = 51)	H/*χ*²	*P*
Gestational age at birth (weeks), M (P25, P75)	30.86 (30.00,31.43)	29.86 (29.43,30.57)	29.29 (28.86,29.86)	60.540	<0.001
Birth weight (g), M (P25, P75)	1,490 (1450,1610)	1,450 (1320,1540)	1,320 (1240,1360)	68.392	<0.001
Postmenstrual age (PMA) at initiation of oral feeding (weeks), M (P25, P75)	33.29 (32.43,34.00)	33.14 (32.29,33.86)	34.29 (33.57,34.71)	30.281	<0.001
Weight at initiation of oral feeding (kg), M (P25, P75)	1.70 (1.66,1.82)	1.68 (1.54,1.80)	1.70 (1.57,1.82)	4.381	0.112
Maternal age (years), M (P25, P75)	32 (29,36)	33 (28,36)	33 (30,36)	0.510	0.775
Sex, *n* (%)				0.382	0.826
Male	30 (44.8)	47 (49.5)	25 (49.0)		
Female	37 (55.2)	48 (50.5)	26 (51.0)		
1-minute Apgar score, *n* (%)				1.257^a^	0.533
≥7	63 (94.0)	87 (91.6)	45 (88.2)		
<7	4 (6.0)	8 (8.4)	6 (11.8)		
Mechanical ventilation, *n* (%)				11.344^a^	0.003
Yes	40 (59.7)	70 (73.7)	47 (92.2)		
No	27 (40.3)	25 (26.3)	4 (7.8)		
Pre-feeding behavioral state, *n* (%)				52.557^a^	<0.001
Low arousal	6 (9.0)	19 (20.0)	27 (52.9)		
Moderate arousal	23 (34.3)	52 (54.7)	20 (39.2)		
High arousal	38 (56.7)	24 (25.3)	4 (7.8)		
Mode of delivery, *n* (%)				0.011	0.995
Vaginal delivery	13 (19.4)	18 (18.9)	10 (19.6)		
Cesarean section	54 (80.6)	77 (81.1)	41 (80.4)		
Twin pregnancy, *n* (%)					
Yes	6 (9.0)	8 (8.4)	2 (3.9)	1.260^a^	0.533
No	61 (91.0)	87 (91.6)	49 (96.1)		
Illness severity (NMI), *n* (%)				20.467	<0.001
<4	58 (86.6)	70 (73.7)	25 (49.0)		
≥4	9 (13.4)	25 (26.3)	26 (51.0)		
Maternal perinatal history, *n* (%)				0.271	0.873
Yes	43 (64.2)	62(65.3)	35(68.6)		
No	24(35.8)	33(34.7)	16(31.4)		

C1, the high-start rapid-rise group; C2, the moderate-start accelerated catch-up group; C3, the low-start slow-rise group.

a Fisher's exact test.

### 3.2 Analysis of overall trends

All 3,195 scheduled feeding sessions (213 infants×5 assessment points×3 sessions) were scored. Both sucking and swallowing were scored as 0 in 37 sessions (1.16%), and swallowing was scored as 0 with a nonzero sucking score in 26 sessions (0.81%). No session was excluded because of incomplete observation. The mean oral feeding ability scores of 213 preterm infants across five assessment time points during the first 14 days after oral feeding initiation are shown in Figure 1. Scores were 22.03 ± 2.95 at Day 1 (T1), 23.28 ± 2.86 at Day 3 (T2), 27.16 ± 3.94 at Day 7 (T3), 30.90 ± 4.32 at Day 10 (T4), and 34.41 ± 3.67 at Day 14 (T5), demonstrating a significant overall upward trend (Wald *χ*^2^ = 3,782.69, *p* < 0.001). Error bars represent standard deviation. The *x*-axis shows time points T1 through T5, and the *y*-axis shows oral feeding ability scores.

**Figure 1:**
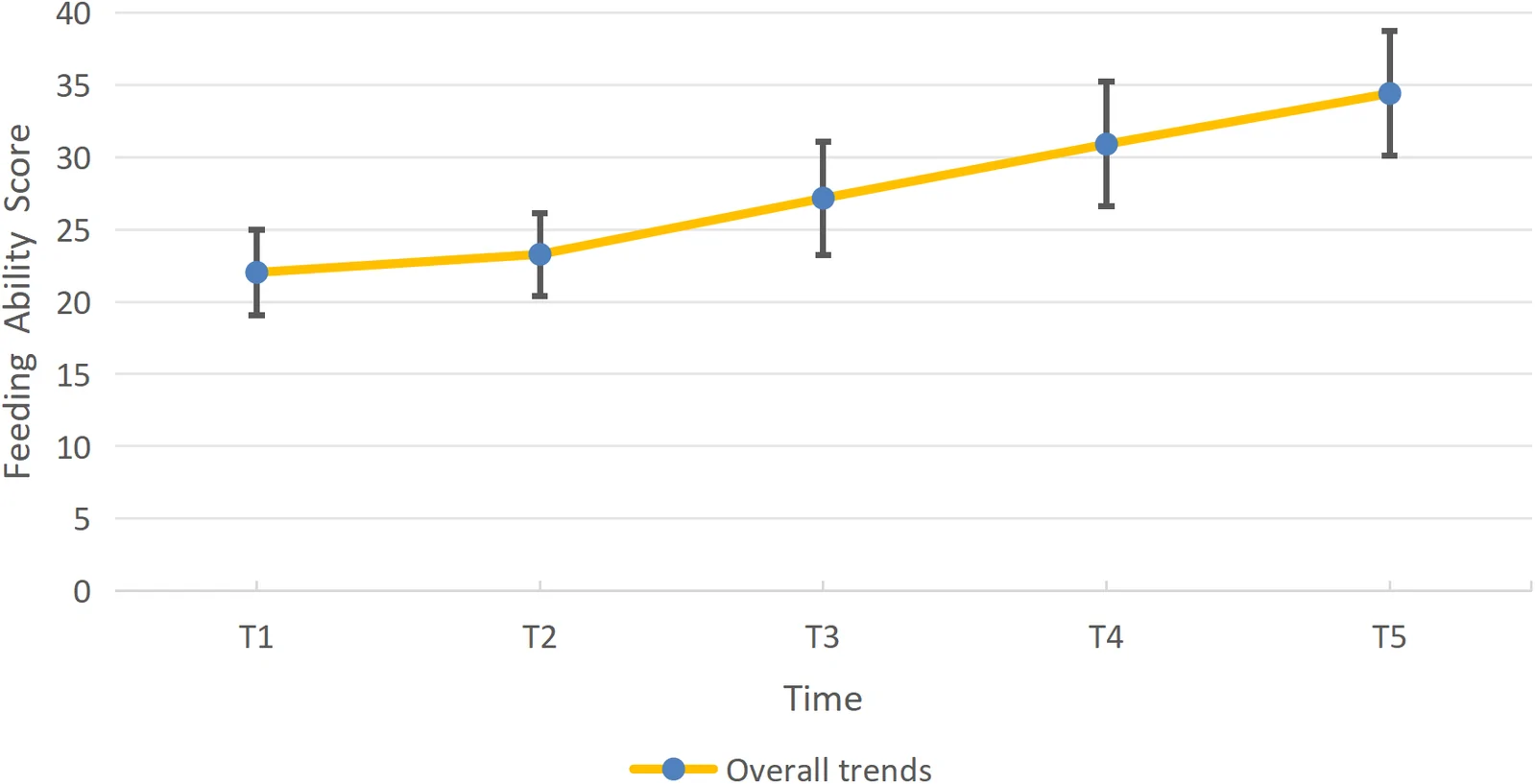
Trends in mean oral feeding ability scores during the first 14 days after oral feeding initiation in 213 preterm infants.

### 3.3 Latent trajectory analysis and naming

Repeated oral feeding ability scores at T1–T5 were analyzed using latent class growth modeling (LCGM). One- to five-class models were fitted sequentially, and model fit indices are presented in Table 2. Compared with the two-class model, the three-class model had lower AIC, BIC, and aBIC values, and the addition of a third class was supported by both VLMR and BLRT. Although the two-class model had higher entropy (0.987 vs. 0.949), both models exceeded the commonly used threshold of 0.80. For the four- and five-class models, AIC, BIC, and aBIC continued to decrease and BLRT remained significant, but VLMR did not support additional classes (*p* = 0.097 and *p* = 0.592), and the additional classes were very small. Considering model fit, class separation, parsimony, clinical interpretability, and class size, the three-class solution was selected.

**Table 2:** Model fit indices for one- to five-class latent class growth models of early oral feeding ability in 213 preterm infants.

Class No.	AIC	BIC	aBIC	Entropy	VLMR (p)	BLRT (p)	Proportion
1	5,837.036	5,863.926	5,838.576	-	-	-	1
2	4,910.270	4,950.605	4,912.581	0.987	0.0000	0.0000	0.319/0.681
3*	**4,680**.**618**	**4,734**.**398**	**4,683**.**699**	**0**.**949**	**0**.**0000**	**0**.**0000**	**0.315/0.446/0.239**
4	4,640.376	4,707.602	4,644.228	0.956	0.0974	0.0000	0.441/0.244/0.296/0.019
5	4,617.534	4,698.205	4,622.156	0.932	0.5920	0.0000	0.441/0.235/0.244/0.019/0.061

*Bold indicates the category we finally selected in the model.

Estimated mean trajectories of oral feeding ability across T1–T5 are illustrated in Figure 2. Class 1 comprised 67 infants (31.5%), with baseline scores of approximately 26 points at T1 that rose steadily to 28 at T2, 32 at T3, 36 at T4, and reached 38 at T5; this trajectory was labeled the “high-start rapid-rise” group. Class 2 comprised 95 infants (44.6%), with baseline scores of approximately 21 points at T1 that increased to 23 at T2, 26 at T3, 30 at T4, and reached 34 at T5; this trajectory was labeled the “moderate-start accelerated catch-up” group. Class 3 comprised 51 infants (23.9%), with baseline scores of approximately 20 points at T1, dipped slightly to 19 at T2, then rose to 23 at T3, 26 at T4, and reached 30 at T5; this trajectory was labeled the “low-start slow-rise” group.

**Figure 2:**
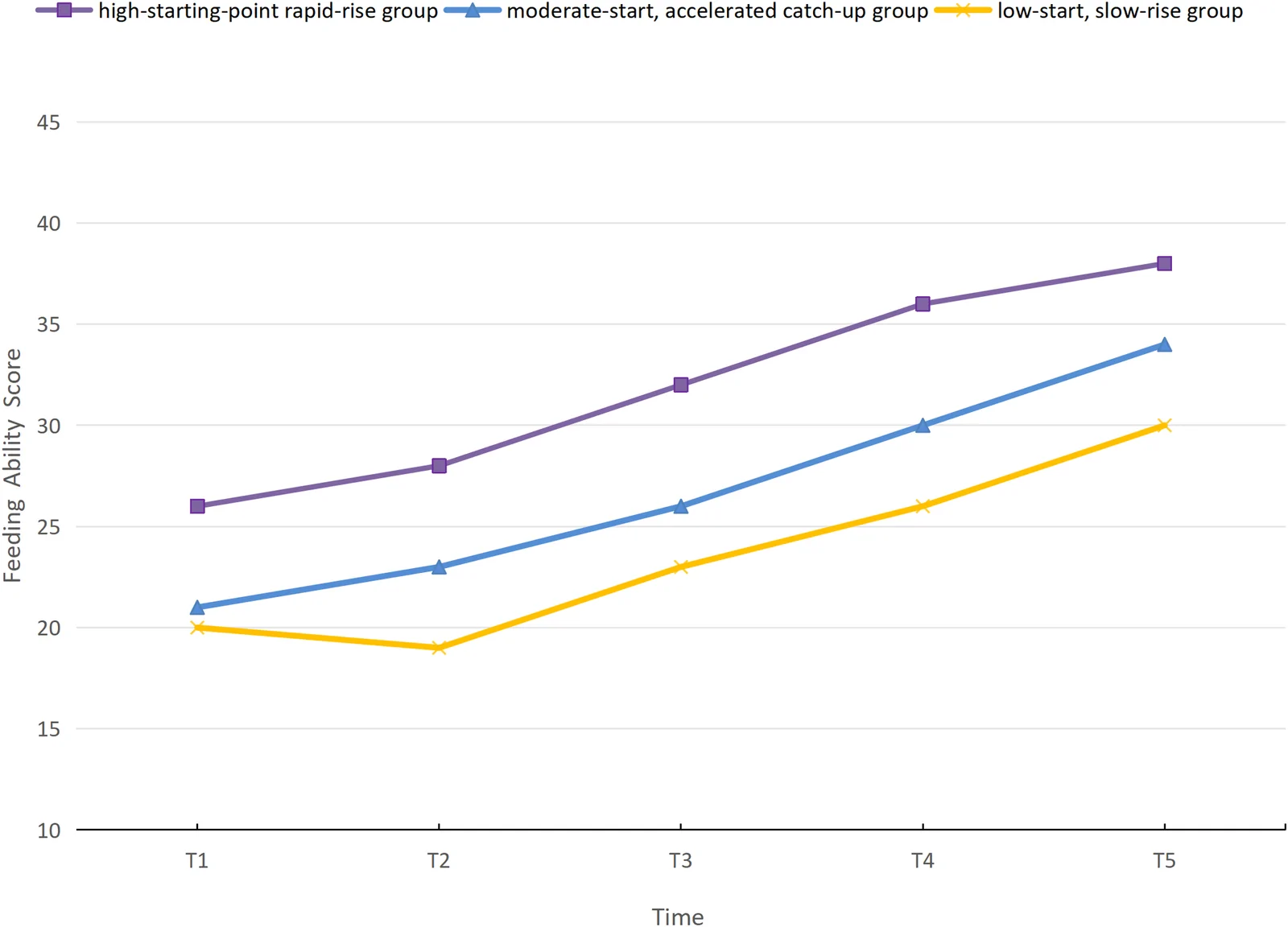
Three distinct developmental trajectories of early oral feeding ability during the first 14 days after oral feeding initiation in 213 preterm infants.

### 3.4 Univariate analysis of oral feeding ability trajectories

Univariate analysis revealed significant differences across the three trajectories in gestational age at birth, birth weight, PMA at oral feeding initiation, mechanical ventilation, illness severity, and pre-feeding behavioral state (Table 1).

### 3.5 Multinomial logistic regression analysis of oral feeding ability trajectories

Multinomial logistic regression was performed with trajectory membership as the dependent variable and the statistically significant variables from univariate analysis as independent variables. The “high-start rapid-rise” group was designated as the reference category. Gestational age at birth, birth weight, and PMA at oral feeding initiation were entered as continuous variables. Mechanical ventilation, illness severity, and pre-feeding behavioral state were treated as categorical variables, with no mechanical ventilation, NMI < 4, and high-arousal state serving as the respective reference categories. All VIF values ranged from 1.085 to 1.288, indicating no serious multicollinearity.

Results are presented in Table 3. For the moderate-start accelerated catch-up group (Class 2 vs. Class 1), lower gestational age at birth, lower birth weight, and low- or moderate-arousal pre-feeding states were independently associated with membership in this group. For the low-start slow-rise group (Class 3 vs. Class 1), lower gestational age at birth, lower birth weight, higher PMA at oral feeding initiation, mechanical ventilation, illness severity NMI ≥ 4, and low- or moderate-arousal pre-feeding states were significantly associated with higher odds of membership in this least favorable trajectory.

**Table 3:** Clinical characteristics associated with membership in early oral feeding ability trajectory classes in multivariable multinomial logistic regression among 213 preterm infants.

Class	Variables	B	SE	Waldc2	P	OR	95% CI
Class 2	Gestational age at birth	−0.917	0.225	16.529	<0.001	0.400	0.257∼0.622
	Birth weight	−0.004	0.001	6.536	0.011	0.996	0.994∼0.999
	Pre-feeding behavioral state (Low arousal)	1.477	0.618	5.716	0.017	4.378	1.305∼14.690
	Pre-feeding behavioral state (Moderate arousal)	1.346	0.419	10.344	0.001	3.843	1.692∼8.729
Class 3	Gestational age at birth	−1.380	0.363	14.437	<0.001	0.252	0.124∼0.513
	Birth weight	−0.013	0.003	25.966	<0.001	0.987	0.982∼0.992
	PMA at initiation of oral feeding	0.815	0.300	7.356	0.007	2.258	1.254∼4.069
	Mechanical ventilation (Yes)	1.584	0.800	3.922	0.048	4.875	1.016∼23.376
	Illness severity (NMI ≥ 4)	1.704	0.705	5.836	0.016	5.497	1.379∼21.911
	Pre-feeding behavioral state (Low arousal)	3.930	0.951	17.067	<0.001	50.908	7.889∼328.497
	Pre-feeding behavioral state (Moderate arousal)	2.776	0.810	11.734	0.001	16.056	3.280∼78.611

Class 1 (C1) was the reference category. For the independent variables, no mechanical ventilation, illness severity < 4, and high-arousal state were the reference groups.

## 4 Discussion

### 4.1 Analysis of trends and trajectories in early oral feeding ability in preterm infants

In this study, oral feeding ability on the first day of oral feeding initiation had a mean score of 22.03 ± 2.95; thereafter, it showed an overall upward trend, with a mean score of 34.41 ± 3.67 by Day 14. Although oral feeding ability showed an overall upward trend during the early stages, LCGM identified three distinct trajectories: the “high-start rapid-rise” group (31.5%), the “moderate-start accelerated catch-up” group (44.6%), and the “low-start slow-rise” group (23.9%). Notably, the “low-start slow-rise” group exhibited the lowest initial oral feeding ability; these infants had a mean score of approximately 30 at Day 14, which was lower than that of the other two groups and may warrant closer monitoring. This group also showed a slight decline in oral feeding ability at T2, which may reflectphysiological immaturity, such as incomplete development of suck–swallow–breathe coordination (24). During initial oral feeding attempts, the inability to coordinate sucking, swallowing, and breathing may predispose these infants to physiological instability, including oxygen desaturation and respiratory pauses, which may compromise oral feeding ability. Given these vulnerabilities, healthcare professionals should prioritize closer monitoring of infants in this group, with particular attention to feeding behavior to detect and prevent physiological instability.

### 4.2 Factors associated with early oral feeding ability in preterm infants

Gestational age and birth weight reflect organ maturity in preterm infants. In this study, infants with higher gestational age and birth weight were more likely to be classified into the “high-start rapid-rise” group, consistent with previous research (25). Conversely, the absence of these protective factors underlies the vulnerability observed in the “low-start slow-rise” group. Premature birth disrupts the critical period for suck–swallow–breathe coordination (24); the abrupt transition from intrauterine to extrauterine environment, coupled with high metabolic demands, renders these infants prone to feeding intolerance and complications. Compared with infants of greater gestational age and higher birth weight, those with lower values exhibit more immature sucking patterns, such as lower maximum sucking pressure and fewer sucks per burst, which may contribute to poorer oral feeding ability (26, 27). Jackson et al. (28) reported that time to first oral feeding and time to full oral feeding decreased with increasing gestational age at birth, and identified gestational age and birth weight as significantly associated with these times. In a cohort of infants born before 30 weeks of gestation, Chinea Jiménez et al. (29) found that lower gestational age at birth and lower birth weight were associated with later postmenstrual age at achievement of full oral feeding. In addition to gestational age and birth weight, mechanical ventilation was also associated with membership in the low-start slow-rise group. Previous studies have linked BPD to altered suck–swallow coordination (30) and prolonged ventilation to later feeding problems (31); however, ventilation duration and BPD were not examined in this study. Because mechanical ventilation is closely related to gestational age and birth weight, this association is best interpreted as a marker of clinical severity rather than an independent causal effect.

An NMI score ≥ 4 was associated with membership in the low-start slow-rise group. Previous studies have reported associations between greater illness severity and delayed attainment of full oral feeding (32) and between respiratory disease and altered suck–swallow coordination (30, 33), consistent with the present findings. However, the NMI is a composite severity measure, and these mechanisms were not directly evaluated in this study; they therefore require prospective verification. Overall, infants with lower gestational age, lower birth weight, higher NMI scores, or mechanical ventilation may warrant closer monitoring and individualized feeding support.

Oral feeding was initiated at a PMA of at least 32 weeks once physiological stability was achieved. Even with this uniform threshold, PMA at initiation was higher in the low-start slow-rise group than in the other two groups (overall *P* < 0.001; Table 1) and remained associated with this trajectory after adjustment (OR = 2.258, 95% CI 1.254–4.069, *P* = 0.007; Table 3), whereas no significant difference was observed between the moderate-start and high-start groups (Wald *χ^2^* = 0.552, *P* = 0.457). Because initiation timing was clinically determined, this finding should be interpreted cautiously: the later initiation in the low-start slow-rise group likely reflected a more adverse clinical profile rather than greater neuromotor maturity (34). A PMA- and stability-based threshold may therefore be a necessary safety criterion, but it may not fully capture behavioral readiness (35, 36). Current international recommendations emphasize individualized, physiologically appropriate feeding management for preterm infants (37), and a recent systematic review reported that cue-based feeding may shorten the time to full oral feeding and reduce feeding intolerance (38). These findings support incorporating behavioral cues into readiness assessment rather than relying on postmenstrual age alone. Such an approach may avoid unnecessary delays for infants who are behaviorally ready to feed while reducing premature attempts in infants who remain drowsy or physiologically unstable. Consistent with this view, pre-feeding behavioral state was associated with trajectory membership in the present study (*P* < 0.001; Table 1): quiet wakefulness before feeding has been related to more efficient oral feeding (39), whereas low- or moderate-arousal states before scheduled attempts were more common in less favorable trajectory groups (40). The difference between Classes 1 and 2 also supports this interpretation: although these groups initiated oral feeding at a similar PMA, Class 2 had more frequent low- or moderate-arousal pre-feeding states and subsequently followed a less favorable trajectory. These findings suggest that behavioral cues should be considered together with physiological stability when assessing readiness for oral feeding. Oral-motor and non-nutritive sucking interventions may improve feeding readiness by stabilizing behavioral state (41). Whether feeding protocols based entirely on behavioral cues, or other structured feeding interventions, can shift infants toward more favorable feeding trajectories remains to be determined in future prospective interventional studies.

### 4.3 Implications for future research

Within a narrow gestational age range and under a standardized feeding protocol, oral feeding ability nonetheless followed three distinct trajectories during the first 14 days after oral feeding initiation. The low-start slow-rise group had a lower mean score than the other groups at the first assessment and continued to show a less favorable course throughout follow-up. Because trajectory membership was determined using all five assessments, this is a retrospective observation and does not establish prospective identification at the first assessment. It suggests that infants assigned to this trajectory may warrant closer monitoring, but prospective identification would require validation in a separate analysis or cohort. In addition, future studies should link trajectory membership to outcomes beyond Day 14, including time to full oral feeding, PMA at attainment of full oral feeding, and length of hospital stay, to establish the clinical relevance of these trajectory groups.

Behavioral state provides a promising starting point for intervention research. Pre-feeding behavioral state was associated with trajectory group membership even though oral feeding was initiated according to postmenstrual age and physiological stability, which is consistent with evidence supporting cue-based readiness assessment (38). Accordingly, future studies should first test whether trajectory classification adds clinical value beyond factors available at feeding initiation, particularly in distinguishing Class 1 from Class 2, and should then compare cue-based with schedule-driven feeding to determine whether feeding outcomes or trajectory assignment can be improved. The low-start slow-rise group, characterized by greater illness severity, mechanical ventilation, later oral feeding initiation, and more frequent low- or moderate-arousal pre-feeding states, offers a suitable population for such trials and for evaluating adjunctive oral-motor or non-nutritive sucking support (35, 41–43).

Nutritional management raises a complementary question. International guidance emphasizes adequate protein and energy intake and individualized human milk fortification (37), yet the present cohort received uniform preterm formula without individualized fortification. Evidence indicates that individualized fortification improves short-term growth (44); whether these effects extend to oral feeding competence can be tested in prospective studies that record actual enteral and parenteral intake and compare nutritional strategies within the same feeding framework.

## Limitations

Several limitations merit consideration. First, this single-center study used a unit-specific feeding protocol consisting of uniform preterm formula, schedule-driven oral feeding attempted regardless of behavioral state, and side-lying positioning, and it enrolled only formula-fed preterm infants without major neonatal comorbidities. Because this feeding protocol was not predominantly cue-based, the observed trajectories may partly reflect the interaction between infant readiness and the feeding protocol rather than feeding maturation alone, and the trajectory patterns and their relative frequencies may not necessarily be reproduced in NICUs using predominantly cue-based feeding protocols. These factors may also limit generalizability to other NICUs and to more medically complex or human-milk-fed infants. Second, follow-up ended on Day 14 after oral feeding initiation, and outcomes beyond this period—time to full oral feeding, postmenstrual age at full oral feeding, and length of hospital stay—were not assessed. Third, behavioral state was recorded immediately before scheduled feeds and may not reflect diurnal variation. Inter-rater reliability was not formally quantified, and these behavioral-arousal categories were not externally validated. Although assessors completed standardized training and were blinded to study hypotheses, these measures do not replace formal inter-rater reliability testing. Therefore, the corresponding behavioral-state findings should be considered exploratory. Fourth, not all variables were measured at the initiation of oral feeding: pre-feeding behavioral state was averaged across all five assessment points, and illness severity was assessed at discharge. These data therefore do not support prospective prediction; the reported associations should be interpreted as exploratory and hypothesis-generating. In addition, despite a standardized protocol, the cohort remained clinically heterogeneous in illness severity, feeding tolerance, and timing of oral feeding initiation. Respiratory variables were limited to a binary history of mechanical ventilation; ventilation duration and respiratory support at oral feeding initiation were not available and could not be adjusted for. Individual daily enteral and parenteral intakes, PN indications and discontinuation, feeding interruptions, and subsequent weight gain were also not recorded. The standardized protocol provided uniform nutritional targets, but actual intake may still have differed across infants, and detailed nutritional management could not be adjusted for in the analyses. Fifth, some multinomial logistic regression estimates were based on sparse cells; for example, only four infants in the low-start slow-rise group were in the high-arousal category, and only four in this group had not required mechanical ventilation. Consequently, confidence intervals were wide; the direction of these associations may still be clinically informative, but their magnitude should be interpreted cautiously. Sixth, because the NMI predates contemporary neonatal care, it may not fully capture current treatment- and practice-related factors. Multicenter studies with prospective nutritional data and longer follow-up are needed to validate the trajectories and their clinical utility.

## Conclusions

This study demonstrates heterogeneity in early oral feeding ability among preterm infants, with three distinct trajectories identified: the “high-start rapid-rise” group, the “moderate-start accelerated catch-up” group, and the “low-start slow-rise” group. Variables associated with trajectory membership included gestational age at birth, birth weight, postmenstrual age at oral feeding initiation, mechanical ventilation, illness severity, and pre-feeding behavioral state. These exploratory findings from a single-center, formula-fed cohort may inform future hypothesis-driven research but require validation in larger, multicenter cohorts before clinical application.

## Data Availability

The raw data supporting the conclusions of this article will be made available by the authors, without undue reservation.
